# The Immediate Effects of Orthoses on Pain in People with Lateral Epicondylalgia

**DOI:** 10.1155/2013/353597

**Published:** 2013-11-19

**Authors:** Ebrahim Sadeghi-Demneh, Fahimehsadat Jafarian

**Affiliations:** Musculoskeletal Research Center, School of Rehabilitation Sciences, Isfahan University of Medical Sciences, Hezarjarib Blvd, Isfahan, Iran

## Abstract

*Objective*. Tennis elbow is a common cause of upper limb dysfunction and a primary reason for pain at the lateral aspect of the elbow. The purpose of this study was to investigate the effects of three commonly used orthoses on pain severity. An elbow band, an elbow sleeve, and a wrist splint were assessed for their ability to reduce the level of reported pain. *Method*. A crossover randomized controlled trial was used. The orthoses were worn in a randomized order, and all participants were required to complete a control trial for which they wore a placebo orthosis. 52 participants with lateral epicondylalgia were recruited, and the level of pain at their elbow was recorded using the visual analogue scale (VAS). *Results*. The reported pain for all orthoses was lower than that of the placebo (*P* < 0.05). Pain reduction was significantly greater with a counterforce elbow band or a counterforce elbow sleeve compared to a wrist splint (*P* < 0.01). There was no significant difference between a counterforce elbow band and a counterforce elbow sleeve (*P* = 0.23). *Conclusion*. All the types of orthoses studied showed an immediate improvement on pain severity in people with lateral epicondylalgia. The counterforce elbow orthoses (elbow band and elbow sleeve) presented the greatest improvement, suggesting that either of them can be used as a first treatment choice to alleviate the pain in people with tennis elbow.

## 1. Introduction

Lateral epicondylalgia, or tennis elbow, is a painful condition associated with repetitive strains of the wrist extensor tendons [1–4]. This overuse syndrome is characterised by pain and tenderness below the lateral epicondyle, which is exacerbated when subjected to a resisted wrist extension [1]. People who engage in the repetitive hand tasks are more susceptible to this type of injury [5–7]. This injury occurs in 1–3% of the general population [8]; however, this increases to more than 50% in tennis players, a population that use regular repetitive hand functions [9]. The cause of the lesion is believed to be the overloading of the wrist extensors' common origin at the lateral aspect of the elbow [3]. 

There are numerous treatment modalities employed for lateral epicondylalgia, including orthotics. The main objective in orthotic therapy is to target the cause of the lesion by reducing the overloading strains on the common origin of the wrist extensors [10, 11]. A number of strategies were reported to achieve this goal and several orthotic approaches have been used accordingly. An elbow band (strap) is a common device which is worn below the elbow. It applies a binding force over the wrist extensor muscle mass at their proximal origin (this is also known as a counterforce orthosis). It has been substantiated, that such a band can reduce the percentage of the elongation and force generation made by musculotendinous fibers above the orthosis [12]. Various types of counterforce elbow orthoses have been developed. Some designs cover a wider area below as well as above the epicondyles and are known as a “sleeve.” Counterforce elbow sleeves are thought to reduce pain more effectively. This is because a wider contact area can stimulate more sensory receptors around the elbow providing a pain alleviation mechanism. An alternative orthotic strategy is to maintain the wrist in slight extension. The wrist extensor muscles act as stabilizers during gripping and other hand functions. Keeping the wrist in extension is thought to have lower physiologic demands on the muscles and therefore reduces the overloading strain on the laden tissues [13–15].

There is conflicting evidence on the effects of orthotic devices on pain management, and therefore, the literature is inconclusive in this field [11, 14]. Some studies have implied a reduction in the pain intensity using an orthosis [16–18], whilst other reports have indicated no significant difference in the pain recorded [19, 20]. The objective of this study was, therefore, to investigate the effects of three types of orthoses on the reported pain of people with lateral epicondyalgia.

## 2. Method

### 2.1. Design

**Figure 1:**
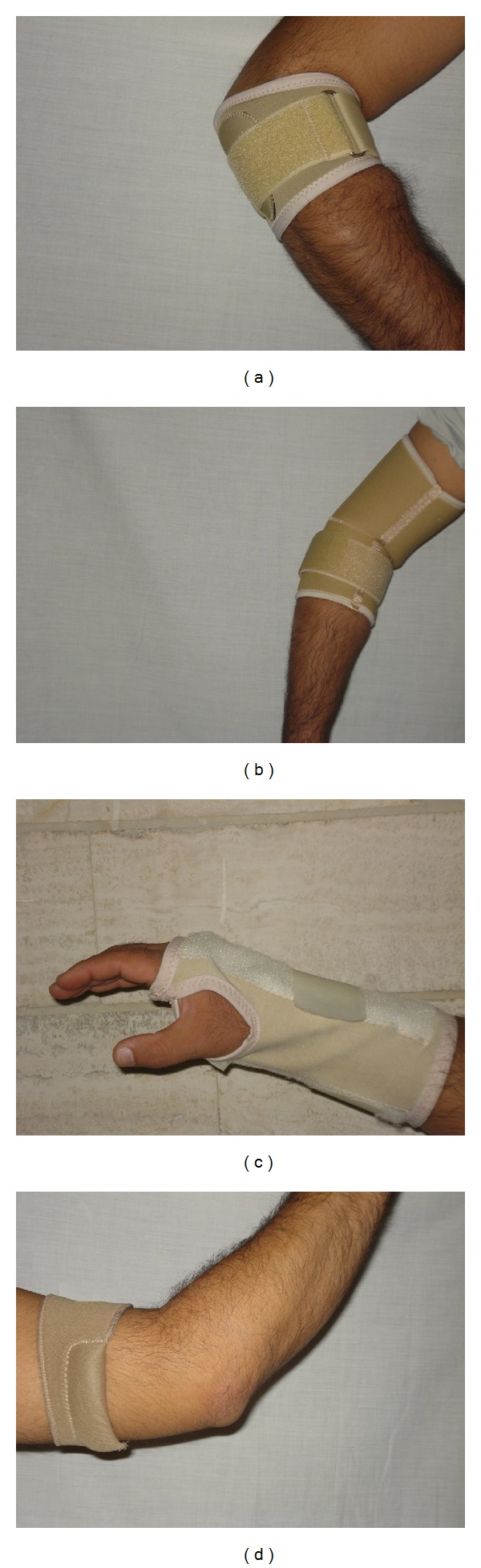
The (a) elbow counterforce band, (b) elbow counterforce sleeve, (c) wrist splint, and (d) placebo.

A randomized controlled crossover trial was used, during which the participants acted as their own controls, and the randomization was provided by the order of orthotic conditions. Three types of orthoses, including a counterforce elbow band, a counterforce elbow sleeve, and an extension wrist elbow splint, were fitted along with a control placebo orthosis (Figure 1). The order of testing was determined by selecting a concealed envelop from a hat. Testing was carried out once orthoses were fitted and the participant felt comfortable. There were approximately 5 minutes between removing one orthosis and fitting the next. Ethical approval was obtained from the ethical committee of Isfahan University of Medical Sciences prior to the start of the study. An informed written consent was obtained from each participant prior to testing. Further details of the testing process are available in the testing protocol, discussed later. 

### 2.2. Participants

People who reported painful symptoms of lateral epicondylalgia for at least 3 weeks prior to testing were recruited for the study. The diagnosis of each participant was made by an orthopaedic specialist, and the participants were referred to a specialist orthotic center (Behgam Clinic, Isfahan, Iran) for providing their orthoses and testings. The definitive diagnosis was based on two criteria: (1) pain and tenderness when palpated below the lateral humeral epicondyle; (2) the pain is aggravated when subjected to a resisted wrist extension. Participants were only included if they tested positive to both criteria. People with a history of surgery, fracture, dislocation, osteoarthritis at the elbow, cervical neuropathy, or previous steroid injection to the elbow were excluded. An optimal sample size of 52 people was calculated from the preliminary data. This data was obtained from a pilot trial of 20 participants, using a power of 0.8 and alpha level at 0.05. 

### 2.3. Orthoses

Three common orthoses for lateral epicondyalgia were compared against a placebo condition. The counterforce elbow band is a 5-cm-wide neoprene band which is fitted around the forearm (1 inch distal to the epicondyle). The elbow band had a pressure pad and a Velcro strap to apply pressure to the forearm muscles. The required size of the band was left to the judgement of the examiner. The band was fitted at the forearm, and the participant made a fist, then strap was tightened. The size of the band was considered suitable if the pressure applied on the forearm was still comfortable after the fist was opened. The counterforce elbow sleeve was made of neoprene rubber and extended 5 inches above and below the elbow joint. A 5-cm-wide Velcro strap was fastened around the forearm, distal to the epicondyles. An appropriate size was decided according to circumferential measurements of 5 cm proximal and distal to the elbow. The extension wrist splint (cock-up) was also made of neoprene and had a rigid polyethylene bar which kept the wrist in 15–20° extension. This position was chosen because it is the optimal position for hand function in people with lateral epicondylalgia [21]. The bar was placed on the palmar surface of the hand and restricted all wrist movements, particularly flexion. The distal trimline of the wrist splint was the metacarpophalangeal crease and splint extended up to two third of the forearm length. The wrist splint had three 2-cm-wide straps used to fasten the splint to the limb. The placebo condition was a 5-cm-wide elastic neoprene, with a 2-cm-wide Velcro strap. The placebo orthosis was fastened circumferentially around the mid-arm but applied no pressure on the origin of the wrist extensor muscles. All treatment and placebo devices were supplied by the same manufacturer (Teknotan Inc., Tehran, Iran).

### 2.4. Testing Protocol

The pain severity of the participants was evaluated after wearing every orthotic or placebo condition, using a 10-cm visual analogue scale (VAS). On this scale, 0 indicated “no pain,” and 10 represented “the most severe pain” [22]. To assess the pain level in each condition, the participant was sat at a table with his/her forearm in pronation and supported by the table, with elbow in 90° of flexion. They were asked to extend the wrist three times and concentrate on their elbow pain. Participants then reported their pain level by drawing a line within 0 to 10 on the scale.

### 2.5. Data Analysis

A one-way repeated measure analysis of variance (ANOVA) was used to compare the reported pain across the testing conditions. If the ANOVA test showed a statistically significant difference, a Bonferroni test was used as a posthoc calculation. The purpose of this was to show pairwise differences among testing conditions. The clinical effectiveness of each condition was compared against others using Cohen's *d* effect size calculation. The effect sizes are defined as small (*d* = 0.2), medium (*d* = 0.5), and large (*d* = 0.8) [23]. The statistical analyses were carried out using SPSS version 17, and the level of significance accepted was at 0.05.

## 3. Results

Fifty-two participants were recruited including 20 men and 32 women. Their mean age was 41.2 ± 8.1 years, and the mean duration of their pain was 18 ± 15 weeks for the men and 15 ± 11 weeks for the women. The right elbow was affected in 35 (70%) of cases, and for 33 of these participants, this was the dominant side. 31 of 32 women were housewives, the remaining one had an administrative job. Twelve (60%) of the men worked in heavy-labor manual occupations, whilst the remaining eight other men had clerical and administrative jobs. The mean and standard deviation (SD) of the reported pain are presented in Table 1. The one-way ANOVA for the reported pain showed a statistically significant difference in orthotic conditions (*P* < 0.001). Posthoc analyses indicated that the pain level was reported less while using either of orthoses (elbow band, elbow sleeve, and wrist splint) compared to the placebo condition (*P* < 0.05) (Table 2). Only posthoc pairwise comparisons between elbow band and elbow sleeve were not significant when comparing the effects of orthotic conditions against each other (*P* > 0.05) (Table 2). 

## 4. Discussion

The results of this study show that an orthosis reduces pain in people with lateral epicondyalgia when assessed immediately after application. Restoring full functionality in the hand is the major goal of lateral epiconylalgia rehabilitation [17]. Many studies used hand grip dynamometry to monitor the effectiveness of their treatment protocol [11, 20, 24, 25]. The measurement of pain intensity is a clinic outcome in the followup of the people with lateral epicondylalgia. Pain quantification does not need special instruments and is a cost-effective method in clinical assessments. Pain estimation using the VAS is an acceptable method to evaluate the hand function in the epicondyalgia condition, which has been shown to have a strong interrelationship with hand grip strength [26]. 

There are several primary techniques of conservative intervention when relieving pain due to tennis elbow, including controlling the inflammation process, promoting healing, local and general muscle strengthening, improvement of soft tissue flexibility, and controlling loads at the lesion area [27]. The use of orthoses has been shown to have superior immediate pain relief and is more acceptable in the daily activities of patients compared to other modalities such as steroids, ultrasound, laser, massage, and exercise therapy [16, 28]. This suggests that an orthosis can be used as an initial therapy and a supportive treatment within other treatment intervals. These pain relief effects seem to be indicative of clinically important changes in the individual function. A reduction greater than 1 cm on the 10 cm VAS has been shown to be in conjunction with an improvement of hand grip strength and a higher user's satisfaction [16, 17, 25, 29]. The conterforce orthoses in the current study (elbow band and elbow sleeve) showed more than 1 cm reduction in the pain level compared to placebo. According to mean differences (MD) and effect size analysis, the elbow band (MD = 1.72 ± 2.3) and elbow sleeve (MD = 1.4 ± 2.26) are both expected to improve the clinical outcomes because they have noticable effect sizes (*d* = 0.74 and *d* = 0.65, resp.) compared to the control condition. In contrast to our findings, others have reported no significant reduction in the pain intensity using counterforce orthoses [19]. There are some differences in the intervention parameters of a past study, which reported no significant effects of bracing [19], and the current study. Although, the placebo conditions were similar in both studies, the strapping systems used to apply the pressure on the wrist extensors appear to be different. Although, the optimal tension of counterforce orthoses has not yet been defined, it has been confirmed that brace tension is associated with pain intensity and functional outcomes in lateral epicondylalgia [30]. The previous study used two 1-cm-wide straps around the forearm, whilst in the current study, compression was applied using a 5-cm-wide strap and a “pressure pad.” The elbow sleeve in the previous trial was made of an elastic material without any forearm strap whilst for our elastic sleeve, a forearm Velcro strap was incorporated as well. This suggests that localizing the pressure on a target area induces a larger binding pressure on the muscles and will have a greater benefit on pain relief.

Our results show that a wrist orthosis is effective at reducing the pain at the lateral epicondyle. In accordance with this finding, many studies had reported similar pain relief effects when the wrist is kept in a resting position with an orthosis [13, 31]. The clinical effectiveness of wrist splint compared to control condition (MD = 0.48 ± 1.69) is questionable, and its effect size is relatively small (*d* = 0.24). A reduction in the pain response was observed in this study, when a counterforce elbow orthosis (band and sleeve) was compared to wrist orthosis (the effect sizes of the comparisons were moderate). There is still conflicting evidence to compare the effectiveness of two major orthotic systems being used for tennis elbow (counterforce orthoses and wrist splints). In contrary to our result, some trials reported no significant difference between the effectiveness of these orthotic systems on pain relief [17, 19, 25]; even one study has suggested that wrist splits are superior in reducing pain [32]. The testing protocol and measurement tools of these studies were substantially different from the current study. In these studies, pain was measured during a number of different hand activities including carrying [32] and hand grip tasks [19, 25] which need a higher level of wrist extensor activity than our study. Furthermore, the pain scale used in one of these studies was not a measure from 0 to 10 [19] and should not be compared with our results. The comparison between elbow band and elbow strap was nonsignificant, and both showed superior improvement over the other conditions. This suggests that either the sleeve or the band can be used to treat the lateral epicondyalgia.

Neither participant nor examiner could be blinded in this study because they could see which orthosis was being fitted. Data collection, however, was a patient-rated method which was not influenced by assessor's judgment. All participants received all the test conditions, and thus, the therapist could not influence the group allocation. The clinical relevance of the findings is uncertain, as this study only assessed the immediate effect of using the orthoses on pain relief. In this study, it has been assumed that if an orthosis is able to address the lesion pathology, its effectiveness should be quickly apparent on pain intensity. Future research should focus on the measurement of the amount of pain perceived by the participants during jobs, daily life, recreation, and sport activities which demand a higher level of wrist extensors contraction. This study did not measure any “wash-out period” for orthotic conditions; forthcoming studies can address any prolonged effect of such orthotic devices after removal. A study on the acceptability to patients and their preference can lead to the development of new designs which would be more competent in the daily life. 

## 5. Conclusion

Three types of orthoses, an elbow band, an elbow sleeve, and a wrist splint, showed an immediate improvement in the pain severity in people with lateral epicondyalgia. The elbow orthoses (sleeve and band) were more effective than the wrist orthosis in relieving pain due to tennis elbow. These findings suggest that orthotic devices can be considered as a therapeutic method for the initial therapy of tennis elbow. No functional outcome was assessed in this study; thus, clinical acceptability of the findings is limited. Attention should also be paid to any adverse effect due to the prolonged use of an orthosis in a treatment plan.

## Figures and Tables

**Table 1:** Descriptive statistics for the expressed pain after wearing each orthosis (according to VAS).

Elbow Band	4.1 ± 2.1
Elbow Sleeve	4.4 ± 1.9
Wrist Splint	5.3 ± 1.6
Placebo	5.8 ± 2.4

Values are presented as Mean ± SD.

**Table 2:** The change in the pain score while wearing different orthoses (according to VAS).

Comparison	Mean difference	*P* value (95% CI)	Effect size
Placebo-elbow band	1.72 (2.3)	0.001*(1 to 2.4)	0.74
Placebo-elbow sleeve	1.4 (2.26)	0.001* (0.75 to 2)	0.65
Placebo-wrist splint	0.48 (1.69)	0.049* ( 0.01 to 0.96)	0.24
Elbow sleeve-elbow band	0.32 (1.86)	0.231 (−0.21 to 51)	0.15
Wrist splint-elbow band	1.24 (2.47)	0.001* (0.53 to 1.94)	0.64
Wrist splint-elbow sleeve	0.92 (2.4)	0.009* (0.24 to 1.6)	0.51

CI means confidence intervals; effect size is calculated using “Cohen's *d*.” *Indicates a statistically significant difference between groups (*P* < 0.05).
